# In Situ Forming Gelatin Hydrogels-Directed Angiogenic Differentiation and Activity of Patient-Derived Human Mesenchymal Stem Cells

**DOI:** 10.3390/ijms18081705

**Published:** 2017-08-04

**Authors:** Yunki Lee, Daniel A. Balikov, Jung Bok Lee, Sue Hyun Lee, Seung Hwan Lee, Jong Hun Lee, Ki Dong Park, Hak-Joon Sung

**Affiliations:** 1 Department of Biomedical Engineering, Vanderbilt University, Nasville, TN 37235, USA; yunki.lee@vanderbilt.edu (Y.L.); daniel.a.balikov@vanderbilt.edu (D.A.B.); jung.bok.lee@vanderbilt.edu (J.B.L.); hj72sung@gmail.com (S.H.L.); 2 Severance Biomedical Science Institute, College of Medicine, Yonsei University, Seoul 120-752, Korea; leeseh@yuhs.ac; 3 Department of Urology, College of Medicine, Yonsei University, Seoul 120-752, Korea; mushroom14@gmail.com; 4 Department of Food Science and Biotechnology, College of Life Science, CHA University, Gyeonggi 443-742, Korea; kdp@ajou.ac.kr; 5 Department of Molecular Science and Technology, Ajou University, Suwon 443-749, Korea

**Keywords:** injectable gelatin hydrogels, patient-derived mesenchymal stem cells, integrin-mediated interactions, material-driven endothelial differentiation, angiogenesis

## Abstract

Directing angiogenic differentiation of mesenchymal stem cells (MSCs) still remains challenging for successful tissue engineering. Without blood vessel formation, stem cell-based approaches are unable to fully regenerate damaged tissues due to limited support for cell viability and desired tissue/organ functionality. Herein, we report in situ cross-linkable gelatin−hydroxyphenyl propionic acid (GH) hydrogels that can induce pro-angiogenic profiles of MSCs via purely material-driven effects. This hydrogel directed endothelial differentiation of mouse and human patient-derived MSCs through integrin-mediated interactions at the cell-material interface, thereby promoting perfusable blood vessel formation in vitro and in vivo. The causative roles of specific integrin types (α_1_ and α_v_β_3_) in directing endothelial differentiation were verified by blocking the integrin functions with chemical inhibitors. In addition, to verify the material-driven effect is not species-specific, we confirmed in vitro endothelial differentiation and in vivo blood vessel formation of patient-derived human MSCs by this hydrogel. These findings provide new insight into how purely material-driven effects can direct endothelial differentiation of MSCs, thereby promoting vascularization of scaffolds towards tissue engineering and regenerative medicine applications in humans.

## 1. Introduction

Over the past few decades, stem cell therapies have demonstrated a certain degree of clinical success for thin tissues such as skin and cartilage [1,2,3]. These favorable outcomes have generated additional enthusiasm that these therapies could be translated to regenerate metabolically active tissues and organs with support from biomaterials. These therapeutic targets, however, require a high degree of vascularization to support influx and outflux of nutrients, oxygen, and waste products because if there are no blood vessels within any 200 µm (maximum) area, cells and tissue undergo necrosis [4]. Without blood vessel formation, stem cell-based approaches will not be fully successful in regenerating damaged tissues, particularly thick tissues, due to limited support for cell viability and desired tissue/organ functionality.

Continuous progress has been made to promote vascularization of scaffolds by incorporating or conjugating vascular growth/signaling factors (e.g., vascular endothelial growth factor; VEGF) [4,5,6,7]. While these approaches are effective in vitro, their in vivo effects are still questionable due to a short half-life (<30 min) of biological molecules under physiological conditions, and side effects associated with hypotension and edema [8,9,10]. Therefore, several methods have been considered in order to increase growth factor bioactivity over extended periods of time such as (i) incorporating components of the normal extracellular matrix (ECM) (e.g., heparin and fibronectin) that stabilize these factors and/or promote their activity; (ii) encapsulating growth factors in protease-resistant reservoirs that serve as a physical barrier against protease attack; and (iii) engineering proteolytic cleavage sites within growth factor proteins to inhibit their degradation [11,12]. However, there are still concerns about the expensive supply of growth factors and paracrine side effects associated with uncontrolled release into systemic circulation.

To address this unmet need, purely scaffold material-driven effects have been explored [13,14,15,16]. Controlling material properties at the cell–material interface (e.g., matrix stiffness and integrin–matrix interactions) can direct stem cell differentiation, which includes endothelial differentiation of mesenchymal stem cells (MSCs) [1,17]. For example, hydrogels have many advantages to mimic tissue properties due to tunable properties, including hydration and stiffness [18,19]. Moreover, their injectable format can provide a 3D microenvironment when cells are encapsulated and delivered to a target site. We previously developed an in situ forming gelatin−hydroxyphenyl propionic acid (GH) hydrogel. This hydrogel type was used as an injectable/sprayable platform upon a horseradish peroxidase (HRP)-mediated cross-linking reaction to deliver therapeutic cells and drugs [20,21,22]. In particular, this gelatin hydrogel, within a specific range of mechanical stiffness (0.5−3 kPa), was found to direct robust differentiation of mouse bone marrow-derived MSCs (mMSCs) into endothelial cells, and induce extensive vasculogenesis without any biological supplementation in vitro and in vivo [23].

In the present follow-up study, we accomplished the following three objectives. First, we identified a mechanism at the cell–matrix interface that is responsible for directed differentiation of mMSCs into endothelial cells. The causative roles of specific integrin types (α_1_ and α_v_β_3_) in directing endothelial differentiation were verified by blocking the integrin functions with chemical inhibitors. Second, to verify the material-driven effect is not species-specific, we confirmed in vitro endothelial differentiation of patient-derived human MSCs (hMSCs) by this hydrogel. Third, we confirmed consequent, improved blood vessel formation of these patient hMSCs when delivered through the gelatin hydrogel system in a subcutaneous implantation model of immune compromised mice. 

While only about ~300 genes out of approximately 20,000 are unique to either humans or mice as revealed from whole genome sequencing, interspecies differences have been rather difficult to predict and understand at times, as evidenced by countless clinical trials that have ultimately failed after successful pre-clinical studies in mice [24]. Therefore, robust validation in human patient-derived MSCs is required for successful translation. To this end, we have isolated bone marrow-derived MSCs from three patients, particularly from old patients (>65 years old) as they stand to benefit the most from regenerative medicine approaches. This approach is necessary to provide new insight into how purely material-driven effects can direct endothelial differentiation of MSCs, thereby promoting vascularization of scaffolds towards tissue engineering and regenerative medicine applications in humans.

## 2. Results and Discussion

### 2.1. Characterization of In Situ Forming Gelatin−Hydroxyphenyl Propionic Acid (GH) Hydrogels

**Figure 1:**
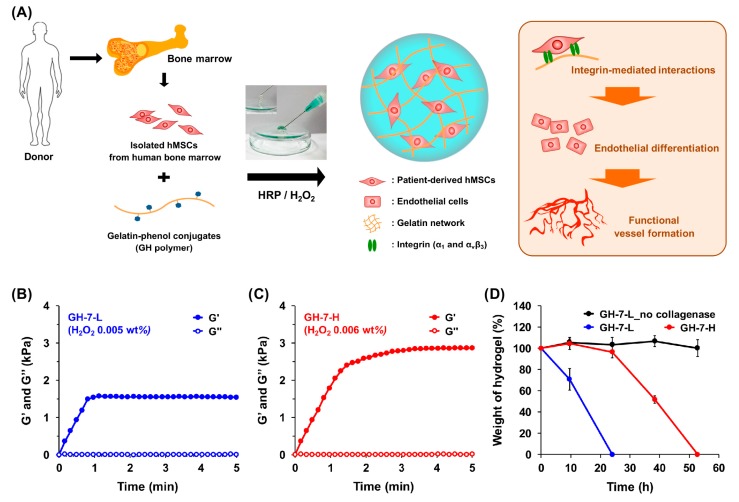
In situ forming gelatin−hydroxyphenyl propionic acid (GH) hydrogels as a translatable platform for mesenchymal stem cell (MSC) delivery. Schematic illustration of GH hydrogels that direct endothelial differentiation of MSCs and induce robust vascularization via integrin-mediated interactions. MSCs are collected from three patients’ bone marrow (>65 years old) and loaded in GH hydrogel matrices during HRP/H_2_O_2_ cross-linking reaction (**A**); Time-sweep elastic modulus (G’) and viscous modulus (G”) of GH hydrogels with different concentration of H_2_O_2_ (0.005 wt % for GH-7-L and 0.006 wt % for GH-7-H) and HRP (2.5 µg/mL) measured by rheometer (**B**,**C**); In vitro degradation profiles of GH-7-L and GH-7-H hydrogels in the presence or absence of collagenase (0.4 µg/mL) treatment (*n* = 3) (**D**).

To prepare in situ cross-linkable gelatin hydrogels, we first synthesized HRP-reactive phenol-conjugated gelatin polymer (GH conjugate, phenolic content = 143 µmol/g of polymer). This GH polymer solution (7 wt %) dissolved in PBS (pH 7.4) was subjected to HRP/H_2_O_2_-tiggered oxidative cross-linking (Figure 1A) [21]. Based upon our previous findings that GH hydrogels with <3 kPa mechanical stiffness led to robust vasculogenic induction of mMSCs in vitro and in vivo, we prepared two different GH hydrogels with distinct degrees of mechanical strength (GH-7-L; 1.4 kPa and GH-7-H; 3.0 kPa) for the present study [23]. The softer hydrogels (<1.0 kPa) were excluded because of fast degradation within a few days in in vitro cell experiments [23].

To characterize the mechanical stiffness of GH hydrogels formed with HRP (2.5 µg/mL) and H_2_O_2_ (0.005 wt % for GH-7-L and 0.006 wt % for GH-7-H), we investigated H_2_O_2_ concentration-dependent viscoelastic modulus (G’ and G”) as a function of time (Figure 1B,C). The time point at which G’ and G” intersect each other (G’ > G”) is defined as a hydrogel formation [25]. The HRP/H_2_O_2_ interactions catalyzed in situ cross-linking among phenolic groups of GH conjugates, and thus resulted in a rapid gelation where an intersected point of G’/G” was observed within 15 s. In addition, higher H_2_O_2_ concentration led to a greater G’ of hydrogels due to the increased cross-linking density where it was confirmed that G’ of GH-7-L and GH-7-H were 1.4 and 3.0 kPa, respectively. We also characterized proteolytic degradation rates of GH-7-L and GH-7-H gels incubated in the media with or without collagenase solution (0.4 µg/mL) as a function of time. As shown in Figure 1D, GH-7-L and GH-7-H hydrogels completely degraded within 24 and 53 h, respectively, whereas the GH-7-L gels incubated in the media without collagenase were stable for 53 h. This result indicates that GH hydrogels can be degraded through the decomposition of gelatin backbone by matrix metalloproteases present in the body, and their degradation rate is dependent on the cross-linking density. 

### 2.2. Gelatin Hydrogel Material-Driven Differentiation of MSCs (Mesenchymal Stem Cells) into Endothelial Cells

We next investigated the interfacial mechanism behind purely gelatin material-driven differentiation of MSCs into an endothelial lineage. While a number of previous studies have shown purely material-driven differentiation of MSCs into osteocytes, neural cells, and chondrocytes, endothelial differentiation most commonly required an extensive use of biochemical agents such as vascular endothelial growth factor (VEGF) and fibroblast growth factor (FGF) [26,27,28]. In our previous study, we also observed an increased expression of VEGF receptor 2 in mMSCs cultured in GH hydrogels without any addition of growth factors [23]. Therefore, we hypothesized that integrins at the cell-matrix interface might play a key role in inducing the endothelial differentiation of MSCs. Integrins are extracellular matrix receptors expressed on the cell membrane, and they serve as a physical anchoring point for adherent cells. Seminal studies have shown that many integrins are responsible for mediating cell–matrix interaction [29,30]. For example, integrins α_1-3_ and α_6_ with their downstream signaling are crucial in driving MSC differentiation to an endothelial lineage by binding to a mixed laminin I and collagen IV substrate. Therefore, inquiring if integrin binding to the GH hydrogel caused endothelial differentiation of mMSCs, as seen in our previous work, would suggest that translation to hMSCs is possible as they express the same integrins.

**Figure 2:**
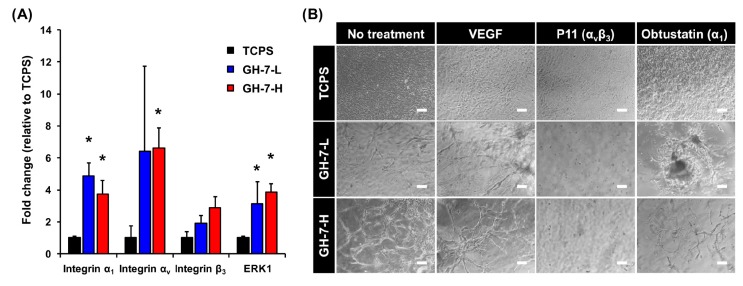
Integrin-mediated mechanisms at the cell–material interface. mRNA expression levels of mMSCs cultured for 15 days either on tissue culture polystyrene (TCPS) (control) or embedded in GH-7-L and GH-7-H hydrogels, * *p* < 0.05 vs. TCPS (*n* = 3) (**A**); Integrin inhibition effects on connectivity of blood vessels formed when mMSCs were cultured in GH-7-L and GH-7-H hydrogels. The experiment groups include mMSCs cultured on TCPS, GH-7-L, and GH-7-H gels for 15 days with no treatment, soluble vascular endothelial growth factor (VEGF), P11 (integrin α_v_β_3_ inhibitor), and obtustatin (integrin α_1_ inhibitor). Scale bars indicate 200 µm (**B**).

In order to identify the types of integrin involved in the material-driven effect on mMSCs, we profiled expression of various integrins through qRT-PCR (Figure 2A). Of 26 integrins known to date, we focused on specific integrins that bind to collagen and other ECM components involved in angiogenesis. Integrin α_1_ (collagen receptor) expression of mMSCs in GH-7-L and GH-7-H gels were significantly upregulated (3.8−4.9 times) compared to the TCPS condition. Additionally, integrin α_v_ and β_3_, together forming a heterodimer that plays a significant role in angiogenesis, were shown to be upregulated. Lastly, ERK1, a key downstream signaling molecule in endothelial differentiation, was significantly upregulated as well. 

We then observed sprouting morphology of mMSCs cultured in GH hydrogels when integrin functions were selectively blocked with chemical inhibitors in order to verify their causative roles for directing endothelial differentiation (i.e., integrin α_v_β_3_ inhibitor; P11 and integrin α_1_ inhibitor; obtustatin). We also included a positive control where soluble VEGF was added in media. As shown in Figure 2B, addition of VEGF improved the degree and connectivity of vasculogenesis in GH hydrogel matrix. However, inhibitor treatment (P11 or obtustatin) attenuated the material-driven effect. P11-treated cells stayed rounded for 15 days, while obtustatin-treated cells tended to aggregate into large clumps with limited vessel sprouting. These results suggest that both integrins α_1_ and α_v_β_3_ are crucial in driving endothelial differentiation of mMSCs in gelatin hydrogels.

### 2.3. In Vitro Endothelial Differentiation of Patient-Derived MSCs in GH Hydrogels

**Figure 3:**
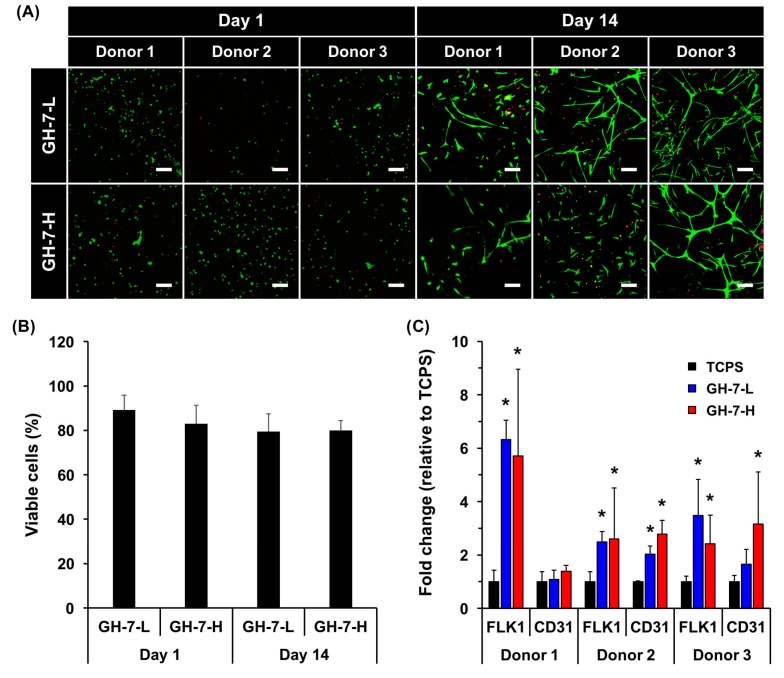
In vitro endothelial differentiation of patient-derived MSCs cultured in GH hydrogels. Live/dead staining images of hMSCs in GH-7-L and GH-7-H gels on days 1 and 14 post culture. Scale bars = 100 µm (**A**); Quantification of viable cells (%) at day 1 and 14 (**B**); mRNA expression levels of endothelial cell markers (FLK1 and CD31) in hMSCs determined by qRT-PCR after 21 days of culture in GH gels. As a control, the same number of cells was seed on TCPS. * *p* < 0.05 vs. TCPS (*n* = 3) (**C**).

Because we confirmed that material-mediated engagement of integrins triggered mMSC differentiation into endothelial cells, we set out to demonstrate translation to hMSCs from representative human donors. Patient-derived hMSCs (donor 1−3; >65 years old-patients), were harvested from bone marrow biopsies, and characterized for purity of isolation. For in vitro experiments, hMSCs were encapsulated in GH gel matrix, and their viability, spreading, and endothelial differentiation of hMSCs were investigated. As seen in Figure 3A,B, live/dead staining images demonstrated excellent survival (>80%) of hMSCs in both GH-7-L and GH-7-H gels throughout the culture period. hMSCs appeared rounded at day 1, but began spreading through the GH matrix over 14 days. Although there was a certain degree of donor-to-donor variation, GH-7-L with lower stiffness revealed better well-elongated and interconnected cell morphology than GH-7-H. The reason why hMSCs cultured in GH-7-L gels spread more is likely due to a faster degradation rate of cross-linked gelatin matrix, which leaves more room for cell spreading and nutrient/oxygen transport. In addition, other previous studies also demonstrated that lower cross-linking density resulted in an increase in mesh size, swelling degree, and degradation rate, thus enhanced cellular activities such as cell migration and growth in 3D cell culture system [31,32].

To verify endothelial differentiation of hMSCs in GH hydrogels, we analyzed gene expression of vascular-endothelial lineage markers (i.e., FLK1 and CD31). For comparison, the same density of cells was also cultured on TCPS as a control. Overall, the expression of endothelial markers in hMSCs cultured in GH gel was significantly upregulated (2.4–6.3-fold for FLK1 and 1.1–3.2-fold CD31, donor 1−3) as compared to TCPS control (Figure 3C). However, no significant difference between GH-7-L and GH-7-H was observed. Taken together, our results demonstrate that the GH hydrogels can promote endothelial differentiation of patient-derived hMSCs in vitro without addition of pro-angiogenic supplements, which is comparable to what was shown with mMSCs [23].

### 2.4. In Vivo Vascularization of hMSCs Delivered with GH Hydrogel

**Figure 4:**
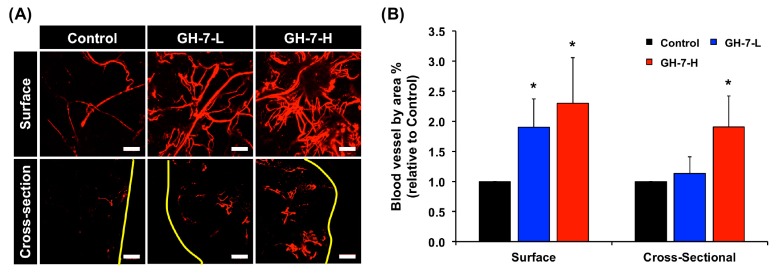
In vivo vascularization of hMSCs with GH hydrogels subcuntaneously delivered into nude mice. Representative images (surface and cross-section) of perfusable vasculature from delivered hMSCs in the GH gel loaded on polyvinyl alcohol (PVA) implants at two weeks post implantation. Yellow lines mark the surface boundaries of implants, and scale bars indicate 200 µm (**A**); Relative ratio of functional blood vessels by crosslinked GH-7-L and GH-7-H gels compared to non-crosslinked GH control (ratio = 1). * *p* < 0.05 vs. Control (*n* = 3, from mixed donor cell groups) (**B**).

Finally, to examine if hMSCs (donor 1−3) in GH hydrogels induced angiogenesis in vivo, we subcutaneously delivered cells in GH gel loaded on PVA scaffolds in immunodeficient mice [23]. The use of immunodeficient mice was necessary in order to avoid severe immune response and eventual rejection of hMSCs derived from unmatched donor to recipient species. Two weeks post-implantation, mice were perfused with a heparinized fluorescent microbead solution for imaging and quantification of perfusable vascular formation. Figure 4A shows fluorescence images of surface and cross-section of scaffolds by fluorescent angiography, and Figure 4B shows the quantification results on relative ratio of blood vessel area % to control. While the non-crosslinked control showed limited vascularization both on the surface and cross-section of the implants, hMSCs in cross-linked GH-7 gels substantially increased vessel formation. In particular, GH-7-H hydrogels most induced blood vessel formation (2.3-fold for surface and 1.9-fold for cross-section, * *p* < 0.05) compared to the control. When GH hydrogels were incubated in vitro with the collagenase as one of the matrix metalloproteinase (MMP) existing in the body, it was found that GH-7-H hydrogels was relatively stable than GH-7-L hydrogel. Accordingly, we speculate that the GH-7-H hydrogel provided more effective biomechanical structure than the non-crosslinked control and GH-7-L gel for cell survival/retention, and consequently supported functional vascularization of hMSCs.

## 3. Materials and Methods 

### 3.1. Materials 

Gelatin (from porcine skin, Type A, >300 bloom), 3-(4-hydroxyphenyl) propionic acid (HPA), 1-ethyl-3-(3-dimethylaminopropyl)-carbodiimide (EDC), *N*-hydroxysuccinimide (NHS), hydrogen peroxide (H_2_O_2_), and horseradish peroxidase (HRP, type VI, 250-330 units/mg solid) were purchased from Sigma-Aldrich (St. Louis, MO, USA). Dimethylformamide (DMF) was obtained from Junsei (Junsei, Tokyo, Japan).

HRP-reactive GH polymer was synthesized by conjugating HPA to the gelatin backbone as previously described [23]. In brief, HPA (20 mmol) was activated with EDC (20 mmol) and NHS (27.8 mmol) in 15 mL of co-solvent (volume ratio of deionized water and DMF = 3:2). After HPA activation for 1 h, the mixture was added to the pre-heated gelatin solution (5 g in 150 mL of deionized water). The reaction was carried out at 40 °C for 24 h. The resulting solution was purified using a dialysis membrane (MWCO = 3.5 kDa) against deionized water for 3 days, and lyophilized to obtain the GH polymer. The conjugated HPA amount of GH polymer was measured by UV–VIS spectrophotometer (V-750, Jasco, Japan) at 275 nm, and determined to be 143 µmol/g of polymer for this study.

### 3.2. Preparation and Characterization of GH Hydrogels with Different Mechanical Stiffness

GH hydrogels were prepared by simply mixing aqueous GH polymer solution in presence of HRP and H_2_O_2_. The pre-heated GH solution (7 wt %) at 40 °C was divided into two aliquots: (1) HRP (2.5 µg/mL) was added to one aliquot, and (2) H_2_O_2_ (0.005 wt % for GH-7-L and 0.006 wt % for GH-7-H) was added to another aliquot (volume ratio of GH:HRP and GH:H_2_O_2_ = 9:1, respectively). Lastly, two aliquots were mixed to generate in situ cross-linked GH hydrogels.

For characterization of gelation kinetics and mechanical stiffness of GH hydrogels formed with different H_2_O_2_ concentrations, the time-sweep elastic (G’) and viscous (G”) modulus of hydrogels (300 µL) were measured at 37 °C for 5 min using an Advanced Rheometer GEM-150-050 (Bohlin Instruments, East Brunswick, NJ, USA) in oscillation mode (strain = 0.01% and frequency = 0.1 Hz, gap = 0.5 mm). 

For in vitro proteolytic degradation testing, GH-7-L and GH-7-H gels were formed in a microtube, and incubated in 1 mL PBS (pH 7.4) with or without 0.4 µg/mL of collagenase (type II, Sigma-Aldrich). At a predetermined time point, the weight of each hydrogel was recorded after removing media, and fresh media was then added for the next time point. The degradation degree of hydrogels was determined by measuring the weight change of initially formed gels with respect to the degraded gels at each time point [20].

### 3.3. In Vitro 3D Culture of hMSC in GH Hydrogels

hMSCs were isolated from the bone marrow of male patients (>65 in age) free from any blood disorders and cancer (hMSC harvest approved by Vanderbilt University Medical Center IRB#150133 February, 2015). FACS was used to isolate hMSCs that are CD14-/CD20-/CD34-/CD73+/CD90+/CD105+. Cells (donor 1−3) were suspended in GH + HRP solution at the concentration of 2 × 10^6^ cells/mL, and this suspension was then mixed with GH + H_2_O_2_ solution to make GH hydrogels incorporating cells. As a control, the same number of cells was also seeded on tissue culture plate (TCPS) without GH hydrogel. After GH hydrogels were formed on the well plate, DMEM supplemented with 10% FBS and 1% penicillin-streptomycin was added, and media was changed every 1–2 days. For the inhibition study, murine MSCs (mMSCs, GIBCO) were used with same conditions and procedures as described above. P11 (EMD Millipore, Billerica, MA, USA) was used at 10 µM, obtustatin (Tocris Biosciences, Bristol, UK) at 10 nM, and mVEGF (Sino Biological Inc., North Wales, PA, USA) at 50 ng/mL as supplements [33,34]. The cellular morphological changes ofcells cultured in GH hydrogels for 15 days were observed by a Nikon Eclipse Ti microscope.

For live/dead staining assay, hMSCs (donor 1–3) cultured in GH-7-L and GH-7-H hydrogels for 1 and 14 days were stained with media containing 1 µg/mL propidium iodide (Sigma-Aldrich, St. Louis, MO, USA) and 1 µM calcein AM (Invitrogen, Carlsbad, CA, USA) for 15 min, and then the live/dead cells were identified using a Zeiss 710 confocal laser microscope.

For quantitative polymerase chain reaction (qRT-PCR) analysis, cells encapsulated within GH hydrogels were homogenized with the Trizol reagent (Life Technologies) mixed with chloroform (volume ratio of Trizol and chloroform = 1:5), and phase-separated by centrifugation (15 min, 4 °C). The aqueous phase containing RNA was isolated with RNeasy columns (Bio-Rad, Hercules, CA, USA) according to the manufacturer’s instructions. cDNA was synthesized using a high-capacity cDNA reverse transcriptase kit (Applied Biosystems, Life Technologies, Foster City, CA, USA), and qRT-PCR was performed with a SYBR Green master mix (Bio-Rad) with 15–20 ng cDNA and 500 nM each of forward and reverse primers, using a CFX Real-Time PCR System (Bio-Rad). The qRT-PCR protocol included: 95 °C for 3 min, followed by 40 cycles of denaturation at 95 °C for 30 s, annealing at 58 °C for 30 s, and extension at 72 °C for 30 s. Expression of each gene of interest was normalized to expression of glyceraldehyde 3-phosphate dehydrogenase (GAPDH) as a housekeeping gene, generating the Δ*C*_t_ value, and expression of 2^−ΔΔ*C*t^ relative to the TCPS control with *n* ≥ 3 biological replicates for each experiment is reported. Primer sequences are listed in Table 1, and only those that showed single, specific amplicons were used for qRT-PCR experiments. 

### 3.4. hMSC Delivery in GH Hydrogels on Polyvinyl Alcohol (PVA) Scaffolds In Vivo

The animal experiment was approved by Vanderbilt Institutional Animal Care and Use Committee (IACUC) in accordance with the NIH Guide for the Care and Use of Laboratory Animals (IACUC number: M1600003 approved on 2 June 2015). GH polymer (7 wt %) and H_2_O_2_ (0.005 and 0.006 wt %) were dissolved in DMEM media as described above, while a constant HRP concentration (2.5 µg/mL) was used for all conditions. The procedures for in vivo animal study are comparable to that previously reported [23]. hMSCs (5 × 10^5^ cells)-containing GH hydrogel solutions in a total volume of 60 µL were loaded on porous PVA scaffolds (6 mm in diameter, Medtronics, Dublin, Ireland). As a control, porous PVA scaffolds loaded with non-crosslinked GH solution containing hMSCs were implanted. The gel-scaffold complexes were then subcutaneously implanted on the ventral side of immunodeficient NU/J mice (male, five-months-old) for two weeks. A longitudinal incision (15 mm) was made on the ventral side of mice, and three different gel-scaffold complexes (donor 1−3) were inserted into individual subcutaneous pockets. The skin incision was closed with sutures.

At two-weeks post implantation, mice were perfused under heavy, near-lethal level of anesthesia with 4% isoflurane in 2 L/min oxygen. First, heparin sulfate (0.1 mg/mL) solution dissolved in PBS was injected into the left ventricle to exsanguinate through the cut inferior vena cava. Mice were then perfused with PBS containing fluorescent micro-beads (Invitrogen) for micro-angiography. Scaffolds were subsequently harvested, and analyzed for angiogenesis by micro-angiography using as described previously [35]. Fluorescence images were obtained using a Zeiss 710 confocal laser microscope. ImageJ software (National Institutes of Health; NIH, USA, Version 1.48) was used for all image preparation and analysis, including z-stacking fluorescence images and quantification.

### 3.5. Statistical Analysis 

All results are expressed as mean ± standard deviation. Comparisons among samples in in vitro and in vivo quantitative analysis were performed by a Student’s *t*-test, and *p* < 0.05 was considered statically significant.

## 4. Conclusions

Based on these results, we suggest in situ cross-linked GH hydrogels as a promising tissue engineering template to promote scaffold vascularization using hMSCs. Not only was it found that the material engaged with integrins to trigger endothelial differentiation, these results adds a substantial value to ongoing research in the field as perfusable vasculature can be generated with hMSCs sourced from older donors who would more likely take advantage of stem cell therapies. A future study will be designed to tune the GH hydrogel system to encapsulate more than one type of stem cell or co-culture MSCs with other somatic cell types for tissue type-specific regeneration with improved vascularization. As seen in Figure 4A, vascular networks were shown to branch out within the core of the GH hydrogel, which is a crucial requirement to maintain metabolically active tissues and organs that could be developed in more advanced co-culture experiments. Therefore, continuing and gradual improvements to the chemical design of the GH hydrogel system remain a long-term goal. In conclusion, the findings reported here illustrate an easy-to-use hydrogel system that can serve as a translatable platform technique for generating stem cell-derived endothelialization for future tissue engineering therapies.

## Figures and Tables

**Table 1:** Primer sequences used for quantitative real-time polymerase chain reaction (qRT-PCR).

Gene	Accession Number	Forward Primer (5′–3′)	Reverse Primer (3′–5′)	Species
*Integrin α_1_*	NM_001033228.3	TCAGTGGAGAGCAGATCGGA	CCCACAGGGCTCATTCTTGT	Mouse
*Integrin α_v_*	NM_008402.3	GTGCCAGCCCATTGAGTTTG	TGGAGCACAGGCCAAGATTT	Mouse
*Integrin β_3_*	NM_016780.2	GCCTGGTGCTCAGATGAGACT	GATCTTCGAATCATCTGGCCG	Mouse
*ERK1*	NM_011952.2	CAACCCAAACAAGCGCATCA	AGGAGCAGGACCAGATCCAA	Mouse
*GAPDH*	NM_001289726	TGAAGCAGGCATCTGAGGG	CGAAGGTGGAAGAGTGGGAG	Mouse
*FLK1*	NM_002253.2	GAGGGGAACTGAAGACAGGC	GGCCAAGAGGCTTACCTAGC	Human
*CD31*	NM_000442.4	CCAAGCCCGAACTGGAATCT	CACTGTCCGACTTTGAGGCT	Human
*GAPDH*	NM_002046.4	GCACCGTCAAGGCTGAGAAC	TGGTGAAGACGCCAGTGGA	Human
